# Alpha-Lipoic Acid Inhibits IFN-γ-Induced PD-L1 Expression in Prostate Cancer Cells and Enhances T-Cell-Mediated Anti-Tumor Cytotoxicity

**DOI:** 10.3390/antiox15040413

**Published:** 2026-03-25

**Authors:** Yi-Jan Hsia, Zhang-Min Lin, Tien-Sheng Tseng, Tz-Chong Chou

**Affiliations:** 1 Dental Department, Taipei Tzu Chi Hospital, Buddhist Tzu Chi Medical Foundation, New Taipei City 23142, Taiwan; 2 Devision of Oral and Maxillofacial Surgery, Taipei Tzu Chi Hospital, Buddhist Tzu Chi Medical Foundation, New Taipei City 23142, Taiwan; 3 Cathay Medical Research Institute, Cathay General Hospital, New Taipei City 22174, Taiwan; 4 Institute of Molecular Biology, National Chung Hsing University, Taichung 402805, Taiwan; 5 Doctoral Program in Microbial Genomics, National Chung Hsing University and Academia Sinica, Taipei 115024, Taiwan; 6 Graduate Institute of Medical Sciences, National Defense Medical University, Taipei 11490, Taiwan; 7 Department of Pharmacology, National Defense Medical University, Taipei 11490, Taiwan; 8 China Medical University Hospital, China Medical University, Taichung 404327, Taiwan

**Keywords:** interferon-γ, prostate cancer, PD-L1, α-lipoic acid, STAT1, c-Myc, oxidative stress

## Abstract

The programmed death-ligand 1 (PD-L1) plays a critical role for promoting cancer immune evasion. However, the resistance to PD-L1-targeted immunotherapy greatly limits its application. α-lipoic acid (ALA) is an endogenous antioxidant, while whether ALA affects PD-L1 expression remains unknown. In IFN-γ-stimulated castration-resistant prostate cancer (CRPC)-mimicking PC3 and DU145 cells, the expression of PD-L1 and its regulatory genes was determined by Western blotting, RT-PCR, and immunofluorescence. The T-cell-mediated tumor-killing activity was evaluated in a co-culture system of cancer cells and Jurkat T cells. ALA significantly inhibits IFN-γ-induced PD-L1 protein and mRNA expression without affecting its degradation. The upstream genes accounting for PD-L1 induction, including JAK1/STAT1/IRF-1 cascade, c-Myc, HIF-1α, and GSK3β activity, were markedly suppressed by ALA. The decreased expression of PD-L1 and these regulators by ALA is also modulated by attenuation of mTOR/p70S6K/4EBP1-dependent protein translation and ROS production. In the co-culture system, ALA markedly increased T-cell-mediated tumor-killing activity compared to that of ALA treatment alone, suggesting that ALA may augment the antitumor immunity. Collectively, we demonstrated that ALA-mediated inhibition of PD-L1 expression is regulated by multiple mechanisms, which indicates that ALA may be a potential agent to enhance cancer immunotherapy, particularly in CRPC.

## 1. Introduction

Immune evasion is a hallmark of tumor, which allows tumor cells to escape recognition and elimination by the host immune system, thereby promoting tumor progression and therapeutic resistance. Programmed death ligand-1 (PD-L1) is an immune checkpoint molecule expressed on cancer cells and plays a pivotal role in tumor immune escape by binding to its receptor programmed death-1 (PD-1), predominantly expressed on T cells. The interaction impairs T cell differentiation and proliferation, leading to attenuation of the cytotoxic activity against tumor cells [1]. It has been reported that increased PD-L1 expression strongly correlated with tumor aggressiveness and poor clinical outcomes in various human cancers, including prostate cancer (PCa) [2]. However, a considerable proportion of cancer patients either fail to respond or develop a resistance to PD-L1/PD-1-targeted immunotherapy, thereby strongly limiting the clinical application. Induction of PD-L1 expression is thought to be a major mechanism causing tumor immune resistance. Thus, suppressing PD-L1 expression is an effective way to enhance the host’s antitumor immunity and attenuate tumor progression [3]. Several oncogenic and inflammatory signaling pathways have been demonstrated to regulate PD-L1 expression at different levels, including transcriptional, translational, and post-translational processes [4]. Interferon-γ (IFN-γ), predominantly secreted by CD8^+^ T cells and other immune cells, binds to its receptor (IFNGR1/2), resulting in the phosphorylation of JAK1/2 and activation of signal transducer and activator of transcription 1 (STAT1). Then, activated STAT1 translocates into the nucleus and induces the transcription of interferon regulatory factor 1 (IRF-1) and subsequent PD-L1 expression via directly binding to the PD-L1 promoter [5,6]. Accordingly, suppressing IFN-γ/JAK1/STAT1/IRF-1 signaling pathway may downregulate PD-L1 expression.

c-Myc is a potent oncogenic transcription factor, promoting cancer cell growth and proliferation [7]. c-Myc also activates PD-L1 transcription in cancer cells via directly binding to the PD-L1 promoter [8]. Conversely, blocking c-Myc activity greatly inhibits PD-L1 expression, further supporting that c-Myc activation induces PD-L1 expression [9]. Under the hypoxic tumor microenvironment that often occurs in solid tumors such as PCa, the hypoxia-inducible factor 1α (HIF-1α), a transcriptional factor, becomes stabilized and activated, which in turn promotes the transcription of several downstream genes involved in angiogenesis, metastasis, metabolism, and cell survival [10,11]. Importantly, HIF-1α can bind to hypoxia-response elements (HREs) in the PD-L1 promoter and induces PD-L1 transcription, thereby facilitating immune evasion and tumor progression [12,13]. Recent studies have confirmed that elevation of reactive oxygen species (ROS) production upregulates PD-L1 expression in cancer cells, whereas the effect can be reversed by ROS scavengers [14,15]. Since ROS are upstream modulators of PD-L1 expression, reduction in ROS production may increase the efficacy of cancer immunotherapy. Taken together, suppressing the interconnected pathways, including the JAK/STAT1/IRF-1 cascade, c-Myc, and HIF-1α, as well as ROS generation represents a promising strategy to reduce PD-L1 expression, which ultimately enhances the effectiveness of cancer immunotherapy.

Alpha-lipoic acid (ALA) is a naturally occurring compound with multiple biological functions, including anticancer and antioxidant activities [16,17,18]. In general, ALA is a safe and well-tolerated compound with minimal adverse effects, making it an attractive candidate for adjunctive cancer therapy [19]. To date, whether ALA affects PD-L1 expression and T-cell-mediated antitumor immunity in IFN-γ-stimulated PCa cells is still unknown. In this study, we examined the effects of ALA on PD-L1 expression in IFN-γ-stimulated advanced PCa cells and T-cell-mediated cytotoxicity and further investigated the molecular mechanisms involved.

## 2. Materials and Methods

### 2.1. Reagents

(±)-α-lipoic acid (T5625) and cycloheximide (C7698) were purchased from Sigma (Burlington, MA, USA). Human IFN-γ (HY-P702), Ruxolitinib (HY-50856), Fludarabine (HY-B0069), IRF1-IN-2 (HY-171007), 10058-F4 (HY-12702), GN44028 (HY-110266), MG132 (HY-13259), phorbol 12-myristate 13-acetate (PMA) (HY-18739), ionomycin (HY-13434), *N*-acetylcysteine (NAC) (HY-B0215), PF-4708671 (HY-15773), Rapamycin (HY-10219), and Laduviglusib (HY-10182G) were purchased from MedChemExpress (South Brunswick Township, NJ, USA).

### 2.2. Cell Culture

Jurkat T cell (Clone E6-1), DU145, and PC3 cell lines were purchased from Bioresource Collection and Research Center (Hsinchu City, Taiwan). Cells were cultured in RPMI 1640 (Gibco, Billings, MT, USA) medium with 2 mM L-glutamine, 1% penicillin–streptomycin–amphotericin B, and 10% FBS (Hyclone, Logan, UT, USA) and maintained in humidified atmosphere of 5% CO_2_ at 37 °C.

### 2.3. Molecular Docking

The LibDock module of Discovery Studio 2021 was used for molecular docking assays to elucidate the binding interactions of ALA with PD-L1. The crystal structure of PD-L1 (Protein Data Bank IDs: 5O4Y and 5C3T) was selected as a receptor model for docking analysis. Prior to docking, ALA was treated with ligand preparation and energy minimization procedures in Discovery Studio 2021, ensuring accurate conformational optimization. Then, the spatial binding orientation and interaction profile of ALA with PD-L1 were investigated.

### 2.4. Cell Fractionation

The nuclear and cytosol fractionation kit (ab289882, Abcam, Cambridge, UK) was used to separate nuclear and cytoplasmic proteins of cells according to the manufacturer’s instruction.

### 2.5. Immunofluorescence Assay

The PCa cells were seeded on coverslips (30,000 cells per coverslip) and treated with IFN-γ and ALA for 6 h. Then, cells were fixed with 4% paraformaldehyde for 15 min, followed by three washes with PBS. After permeabilization with 0.2% Triton X-100 for 15 min, cells were incubated with primary antibodies (1:100) at 4 °C overnight. After three additional PBS washes, the cells were incubated with corresponding secondary antibodies (1:250) for 1 h at room temperature and subsequently stained with DAPI (40, 6-diamidino-2-phenylindole) for 5 min. The fluorescence images were photographed using a THUNDER microscope (Leica, Wetzlar, Germany).

### 2.6. Western Blot

The cells were lysed in RIPA lysis buffer (89900, ThermoFisher, Waltham, MA, USA) with protease and phosphatase inhibitors. Protein concentration of lysate was quantified using BCA assay kit (23225, ThermoFisher), and then the lysate was mixed with 1× Laemmli dye and beta-mercaptoethanol. After being boiled at 98 °C, 10 μg of protein lysate was loaded per lane and resolved with 7.5% or 10% SDS-PAGE for 150 min on ice. Next, the proteins on gel were transferred onto polyvinylidene difluoride membrane and blocked in 5% non-fat milk in TBST for one hour at room temperature. After washing with TBST, the membrane was incubated with primary antibody at 4 °C overnight. Following washing with TBST thrice, membrane was incubated with HRP conjugated secondary antibody (111-035-003, Jackson ImmunoResearch, West Grove, PA, USA) for one hour at room temperature. The blot was visualized using chemiluminescence and detected by TOPBIO MultiGel-21 (TOPBIO, New Taipei City, Taiwan). The band intensity was analyzed by ImageJ (version 1.53v) (National Institutes of Health, Bethesda, MD, USA). The following primary antibodies were purchased from Cell Signaling Technology (Danvers, MA, USA): p-JAK1 Tyr1034/1035 (74129, 1:1000), JAK1 (3344, 1:1000), STAT1 (14994, 1:1000), IRF-1 (8478, 1:1000), mTOR (2983, 1:1000), p-p70S6K Thr389 (9205, 1:1000), p70S6K (9202, 1:1000), 4EBP1 (9644, 1:1000), GSK3β (9315, 1:1000), PARP (9532, 1:1000). The following antibodies were purchased from ProteinTech (Rosemont, IL, USA): HIF-1α (20960-1-AP, 1:1000), GAPDH (60004-1-Ig, 1:5000). Antibody specific to p-STAT1 Tyr701 (A16463, 1:1000) was obtained from Antibodies (Cambridge, UK). The following antibodies were purchased from GeneTex (Hsinchu City, Taiwan): p-GSK3β Ser9 (GTX86837, 1:2000), c-Myc (GTX103436, 1:2000), PD-L1 (GTX104763, 1:2000), vinculin (GTX113294, 1:2000), and p-4EBP1 Thr37/46 (GTX133181, 1:1000). The PARP, vinculin, and GAPDH were used as internal loading control.

### 2.7. RNA Extraction and Real-Time Quantitative Polymerase Chain Reaction (RT-qPCR)

The total RNA of cells was extracted using GENEzol™ TriRNA Pure Kit (GZXD100, Geneaid, New Taipei City, Taiwan) following manufacturer’s instructions. RNA was reverse-transcribed by HiSenScript™ RH(-) RT PreMix Kit (25087, iNtRON Biotechnology, Seongnam-si, Gyeonggi-do, Republic of Korea). The RT-qPCR was performed with QuantStudio 5 real-time PCR system (ThermoFisher) and PowerUp™ SYBR™ Green Master Mix (A25742, ThermoFisher). qPCR primer pairs were (5′-3′): *CD274* (F: TGCCGACTACAAGCGAATTACTG) and (R: CTGCTTGTCCAGATGACTTCGG) (NM_014143.4), and *ACTB* (F: CACCATTGGCAATGAGCGGTTC) and (R: AGGTCTTTGCGGATGTCCACGT) (NM_001101.5). *ACTB* was used as the reference gene.

### 2.8. Intracellular ROS Measurement

DCFDA (10 μM) (ab273640, Abcam) in serum-free medium was incubated with cells for 1 h at 37 °C. Then, DCFDA was discarded, and the fluorescence intensity was measured in PBS using CLARIOstar Plus (BMG Labtech, Ortenberg, Germany) (Ex/Em = 495/529 nm).

### 2.9. T-Cell-Mediated Antitumor Cytotoxicity in Co-Culture of Jurkat T Model

PCa cells were treated with indicated regents for 24 h, and the culture medium was replaced. Jurkat T cells were activated by PMA (50 ng/mL) and ionomycin (1 µg/mL) for 24 h. Then, activated Jurkat T cells were added and co-cultured with PCa cells at a ratio of Jurkat T:PC3 (4:1) or Jurkat T:DU145 (1:1) for 24 h. Then, the suspended Jurkat T cells were removed, and the remaining PCa cells were stained by 0.1% crystal violet for 10 min followed by washing three times and imaged. The cell viability of PCa cells was determined by CCK-8 assay (HY-K0301, MedChemExpress).

### 2.10. Statistical Analysis

All experiments were conducted at least three times. The data were expressed as mean ± S.E.M. The difference between treatments was analyzed by one-way ANOVA and Dunnett’s two-tailed test. Differences were considered statistically significant when *p* < 0.05.

## 3. Results

### 3.1. ALA Inhibits IFN-γ-Induced PD-L1 Expression

**Figure 1:**
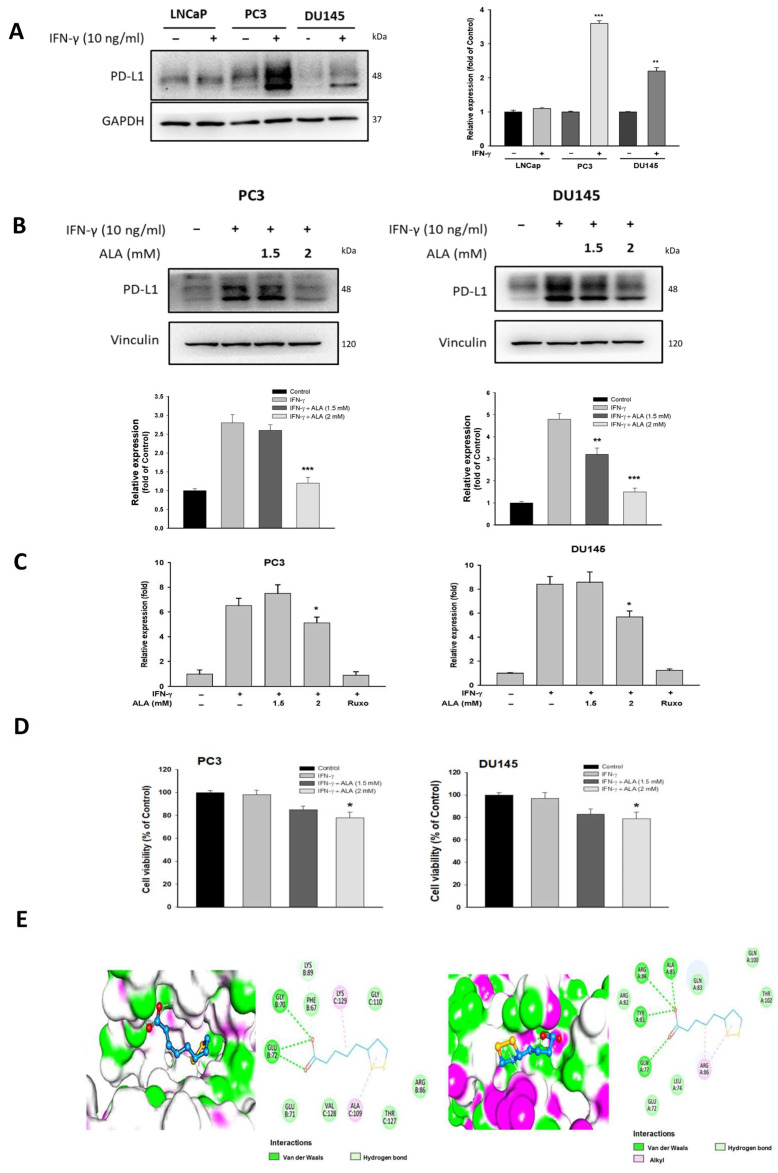
ALA inhibits IFN-γ-induced PD-L1 expression in various PCa cells. (**A**) The LNCap, PC3, and DU145 cells were treated with IFN-γ (10 ng/mL) for 6 h. The PD-L1 expression was determined. Results were expressed as mean ± S.E.M. ** *p* < 0.01, *** *p* < 0.001 vs. respective control. (**B**,**C**) The PC3 and DU145 cells were pretreated with ALA (1.5, 2 mM) or Ruxo (0.5 μM) for 1 h, followed by addition of IFN-γ for 6 h, and the protein expression and mRNA of PD-L1 were determined. (**D**) The effects of treatment with ALA (1.5, 2 mM) for 24 h on the cell viability of IFN-γ-stimulated PC3 and DU145 cells were examined. (**E**) The binding of ALA to PD-L1 was evaluated by the molecular docking assay. Results are expressed as mean ± S.E.M. * *p* < 0.05, ** *p* < 0.01, *** *p* < 0.001 vs. IFN-γ-treated alone cells.

There are two different types of human PCa cells. The androgen-independent PCa cell lines (PC3 and DU145) exhibit more aggressive and metastatic characteristics than androgen-sensitive PCa cell line (LNCap). The PC3 and DU145 cell lines are widely used as standard in vitro models for advanced metastatic castration-resistant PCa (CRPC) research. In response to IFN-γ (10 ng/mL), the PD-L1 expression in PC3 and DU145 cells was higher than that of LNCaP cells (Figure 1A), suggesting that PC3 and DU145 cells may exhibit greater resistance to immunotherapy. Thus, we chose PC3 and DU145 cells for subsequent experiments. Previous studies have demonstrated that ALA at concentrations ranging from 0.5 to 2.5 mM exhibits a significant anticancer effect in different cancer cell types [20,21]. Our results revealed that ALA (2 mM) significantly inhibited the protein and mRNA expression of PD-L1 in IFN-γ-stimulated PC3 and DU145 cells (Figure 1B,C). As expected, the PD-L1 mRNA was markedly repressed by Ruxolitinib (Ruxo, 0.5 μM), is a JAK1/2 inhibitor, having an inhibitory effect on PD-L1 expression, and acted as a positive control [22] (Figure 1C). Consistently, ALA (2 mM) significantly reduced the cell viability of IFN-γ-stimulated PC3 and DU145 cells (Figure 1D). In additional experiments, ALA exhibited greater anticancer activity at a higher concentration (4 mM), suggesting a dose-dependent cytotoxic effect on tumor cells. The molecular docking assay showed that ALA had an interaction with PD-L1 (PDB ID: 5O4Y) through residues E72, G70, K129, and A109. ALA also interacted with PD-L1 (PDB ID: 5C3T) through residues N77, Y81, R84, A85, and R86 (Figure 1E), suggesting that ALA may hinder PD-L1-mediated responses via binding to PD-L1.

### 3.2. ALA Inhibits JAK1/STAT1/IRF-1 Signaling Pathway and c-Myc Expression

**Figure 2:**
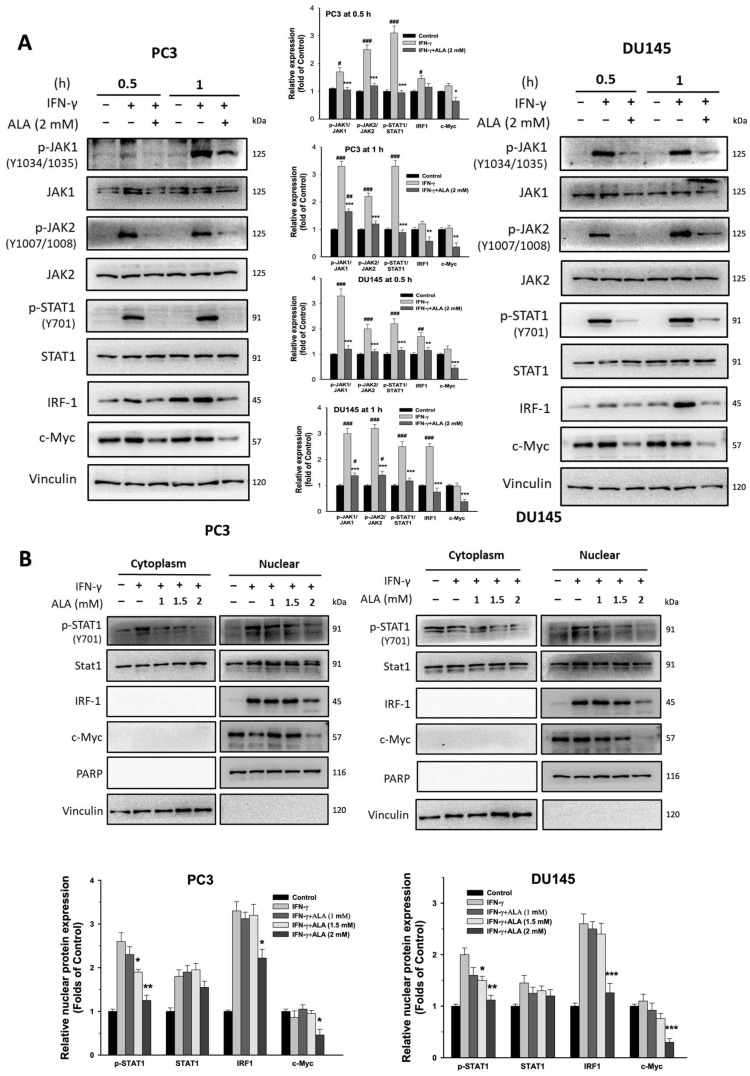
Effect of ALA on JAK1/STAT1/IRF-1 cascade and c-Myc expression. (**A**) The PC3 and DU145 cells were pretreated with or without ALA (2 mM) for 1 h, and then incubated for 0.5 or 1 h in the presence or absence of IFN-γ. The expression of these target genes was determined. (**B**) The PC3 and DU145 cells were pretreated with or without ALA (1~2 mM) for 1 h and further incubation for 6 h in the presence or absence of IFN-γ. The levels of cytoplasmic and nuclear p-STAT1, STAT1, IRF-1, and c-Myc were determined. Results are expressed as mean ± S.E.M. * *p* < 0.05, ** *p* < 0.01, *** *p* < 0.001 vs. IFN-γ-treated alone cells. ^#^ *p* < 0.05, ^##^ *p* < 0.01, ^###^ *p* < 0.001 vs. control group.

**Figure 3:**
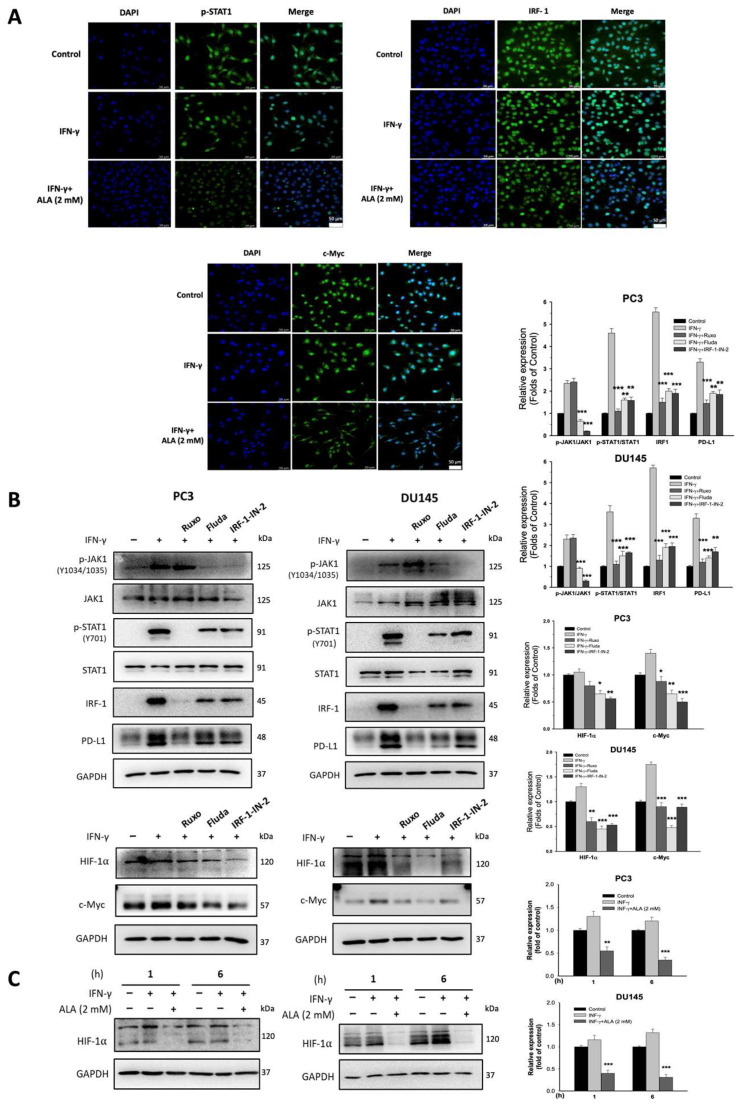
ALA affects PD-L1 expression through JAK1/STAT1/IRF-1 signaling pathway. (**A**) PC3 cells were pretreated with or without ALA (2 mM) for 1 h, and further incubation for 6 h in the presence or absence of IFN-γ. The immunohistochemical assay was used to detect p-STAT1, IRF-1, and c-Myc distribution in cells (scale bar: 50 μm). (**B**) The PC3 and DU145 cells were pretreated with or without Ruxo (0.5 μM), Fluda (1 μM), or IRF-1-IN-2 (20 μM) for 1 h and then cultured with or without IFN-γ for 6 h. The expression of PD-L1 and related target gene was determined. (**C**) The PC3 and DU145 cells were pretreated with or without ALA (2 mM) for 1 h and incubated for 1 or 6 h in the presence or absence of IFN-γ. The expression of HIF-1α was determined using Western blot. Results are expressed as mean ± S.E.M. * *p* < 0.05, ** *p* < 0.01, *** *p* < 0.001 vs. IFN-γ-treated alone cells.

The IFN-γ-activated JAKs/STAT1/IRF-1 signaling pathway and c-Myc and HIF-1α-regulated processes are considered to play a central role in the regulation of PD-L1 expression in cancer cells [23]. Treatment with ALA (1.5 and 2 mM) for 0.5 or 1 h greatly inhibited the phosphorylation of JAK1, JAK2, and STAT1 and the levels of IRF-1 and c-Myc (Figure 2A). Moreover, the nuclear accumulation of p-STAT1, IRF-1, and c-Myc was dose-dependently reduced by ALA (1–2 mM) compared to that of IFN-γ-stimulated PC3 and DU145 cells (Figure 2B). Consistently, the immunofluorescence assays revealed that the nuclear amounts of p-STAT1, IRF-1, and c-Myc were attenuated by ALA (2 mM) in PC3 cells (Figure 3A). 

### 3.3. The Role of JAK1/STAT1/IRF-1 Cascade on PD-L1 Expression

As shown in Figure 3B, blocking JAKs activity by Ruxo strongly inhibited the expression of p-STAT1, IRF-1, and PD-L1. Treatment with Fludarabine (Fluda), a STAT1 inhibitor, also significantly reduced the expression of p-JAK1, IRF-1, and PD-L1. In the presence of IRF-1-IN-2, an IRF-1 inhibitor, the levels of p-JAK1, p-STAT1, and PD-L1 were also markedly repressed, suggesting that a mutual regulation exists in the cascade. Of note, suppressing the JAK1/STAT1/IRF-1 pathway inhibited the expression of HIF-1α and c-Myc (Figure 3B).

### 3.4. The Effects of c-Myc and HIF-1α on PD-L1 Expression

**Figure 4:**
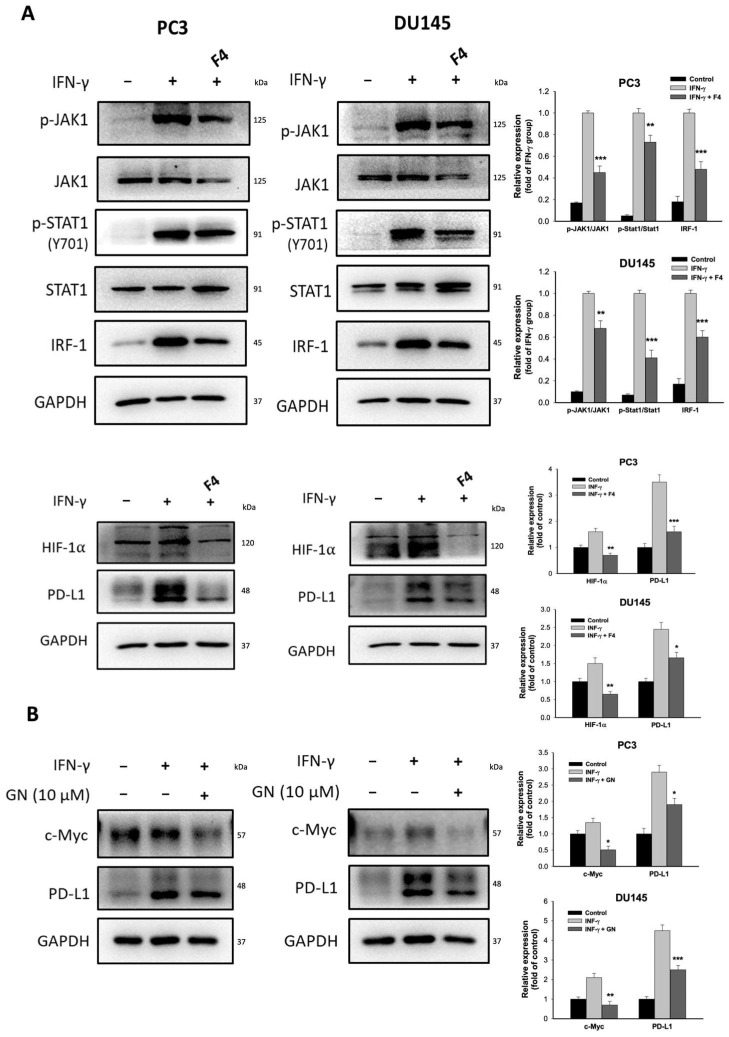
The effects of c-Myc and HIF-1α on the expression of PD-L1 and relative regulators. (**A**) The PC3 and DU145 cells were pretreated with or without 10058-F4 (F4) 80 μM for 1 h and then cultured in the presence or absence of IFN-γ for 6 h. The levels of these target proteins were determined. (**B**) The PC3 and DU145 cells were pretreated with or without GN44028 (GN) 1 μM for 1 h followed by addition of IFN-γ or not. The expression of c-Myc and PD-L1 was determined. Results are expressed as mean ± S.E.M. * *p* < 0.05, ** *p* < 0.01, *** *p* < 0.001 vs. IFN-γ-treated alone cells.

Given that ALA represses c-My expression, the role of c-Myc on the expression of PD-L1 and its upstream regulators was examined. Treatment with 10058-F4 (F4), a c-Myc inhibitor, significantly reduced the p-JAK1/p-STAT1/IRF-1 cascade, as well as the expression of HIF-1α and PD-L1 in IFN-γ-stimulated PC3 and DU145 cells (Figure 4A). Of note, HIF-1α expression was also inhibited by ALA (2 mM) (Figure 3C). In response to GN44028 (GN), an inhibitor of HIF-1α, the expression of c-Myc and PD-L1 was significantly reduced (Figure 4B), suggesting that there is a crosstalk between c-Myc and HIF-1α. Taken together, the inhibition of PD-L1 expression by ALA may be mediated by suppressing p-JAK1/p-STAT1/IRF-1 signaling pathway and the expression of c-Myc and HIF-1α.

### 3.5. Effects of ALA on PD-L1 and IRF-1 Degradation

**Figure 5:**
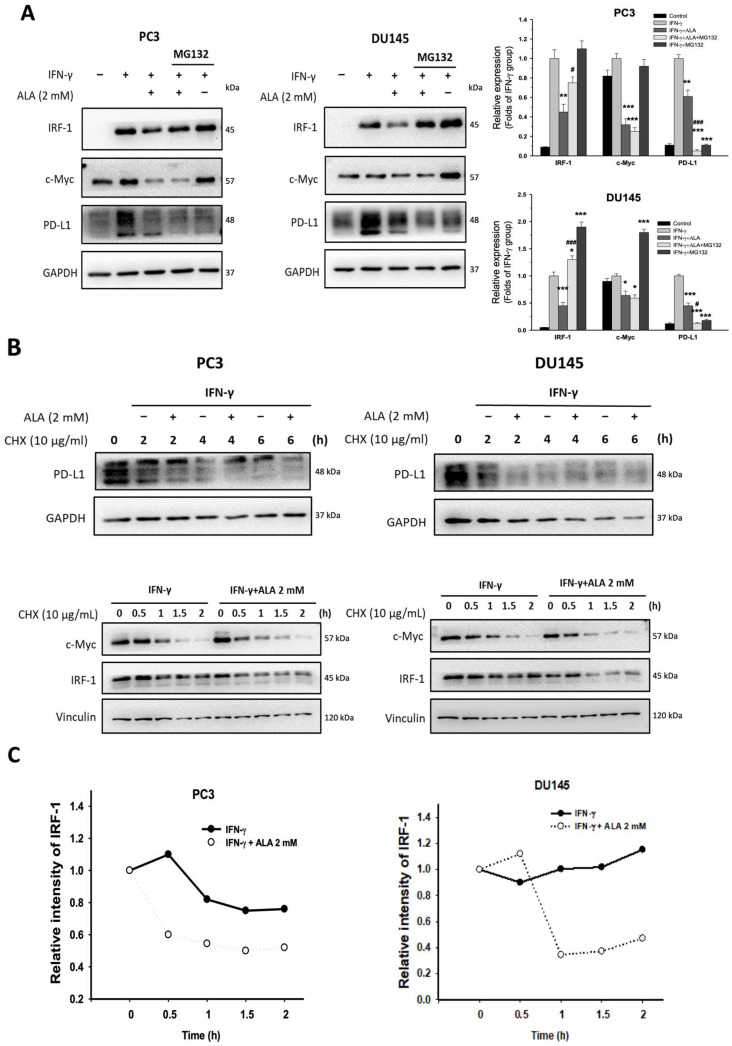
Effects of ALA on the degradation of PD-L1 c-Myc and IRF-1. (**A**) The PC3 and DU145 cells were pretreated with ALA (2 mM) in the presence or absence of MG132 (5 μM) for 1 h and incubated for 6 h with or without IFN-γ. The expression of IRF-1, c-Myc, and PD-L1 was determined. (**B**,**C**) The PC3 and DU145 cells were pretreated with or without cycloheximide (CHX) 10 μg/mL and ALA (2 mM) for indicated time in the presence of IFN-γ. The levels of PD-L1, c-Myc, and IRF-1, as well as their degradation rate, were determined. Results are expressed as mean ± S.E.M. * *p* < 0.05, ** *p* < 0.01, *** *p* < 0.001 vs. IFN-γ-treated alone cells. ^#^ *p* < 0.05, ^###^ *p* < 0.001 vs. IFN-γ and ALA-treated alone cells.

Generally, the cellular protein amounts are primarily controlled by the balance between protein synthesis and degradation. It is well known that IRF-1 and c-Myc are key transcription factors driving PD-L1 expression in cancer cells [9,24]. Next, we examined whether ALA influences the degradation of IRF-1, c-Myc, and PD-L1. In the presence of MG132 (5 μM), a proteasome inhibitor blocking protein degradation, the PD-L1 and c-Myc protein levels were not significantly accumulated, whereas the protein level of IRF-1 increased compared to cells treated with IFN-γ and ALA (2 mM) alone (Figure 5A). Consistently, upon treatment with cycloheximide (CHX), an inhibitor of de novo protein synthesis, ALA (2 mM) did not affect PD-L1 and c-Myc degradation (Figure 5B). However, in response to CHX, the protein levels of IRF-1 were reduced by ALA compared to that of cells treated with IFN-γ alone, indicating that ALA can accelerate IRF-1 degradation (Figure 5B,C). Thus, ALA-mediated reduction in PD-L1 expression may, at least partially, result from impaired protein synthesis, while ALA promotes IRF-1 degradation.

### 3.6. The Effects of ALA on mTORC1/p70S6K/4EBP1 Signaling Pathway

**Figure 6:**
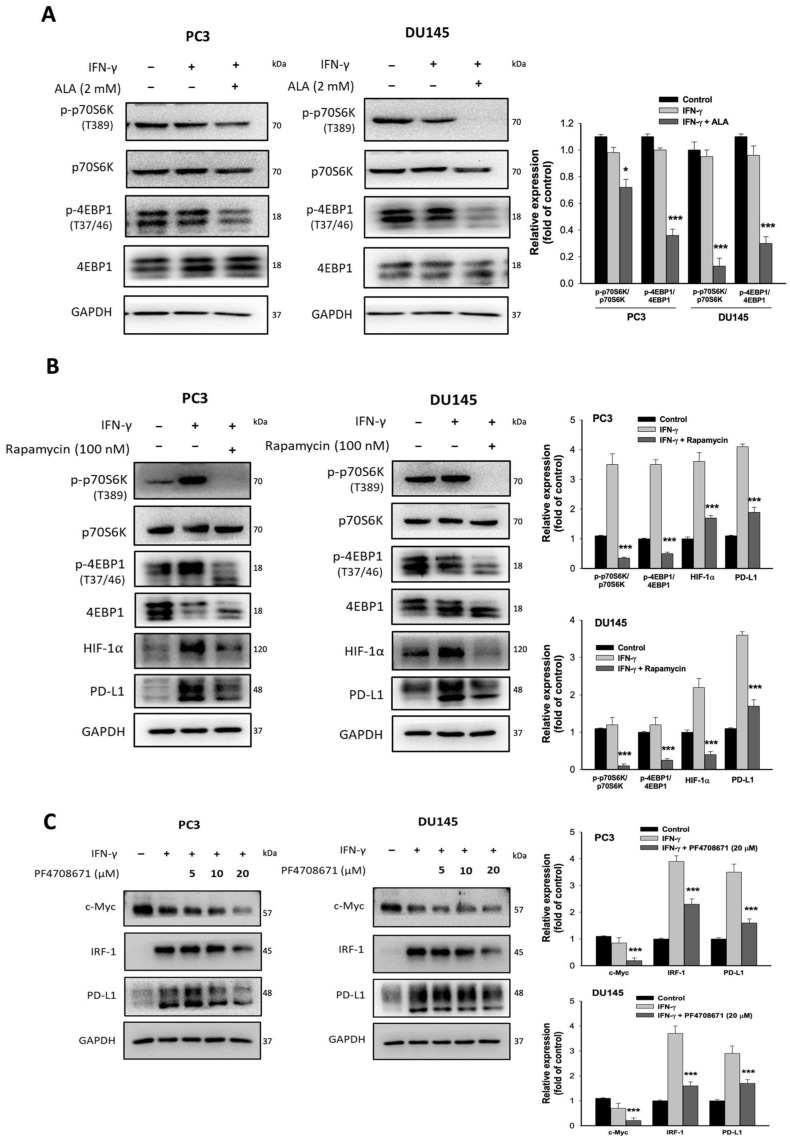
Effects of ALA on mTORC1 and p70S6K-mediated responses. (**A**) The PC3 and DU145 cells were pretreated with or without ALA (2 mM) for 1 h and then incubated for 6 h in the presence or absence of IFN-γ. The expression of p70S6K and 4EBP1 was determined. (**B**) The PC3 and DU145 cells were pretreated with or without Rapamycin (100 nM) for 1 h and then incubated for 6 h in the presence or absence of IFN-γ. The levels of related genes were determined. (**C**) The PC3 and DU145 cells were pretreated with or without PF4708671 (5~20 μM) for 1 h and then incubated for 6 h in the presence or absence of IFN-γ. The levels of c-Myc, IRF-1, and PD-L1 were determined. Results are expressed as mean ± S.E.M. * *p* < 0.05, *** *p* < 0.001 vs. IFN-γ-treated alone cells.

The mechanistic target of rapamycin complex 1 (mTORC1) has been demonstrated to promote ribosomal biogenesis and protein translation by phosphorylating 70 kDa ribosomal protein S6 kinase (p70S6K), a reliable marker of mTORC1 activity. In addition, mTORC1 inhibits translational repressor eukaryotic translation initiation factor 4E binding protein 1 (4EBP1) through phosphorylation of 4EBP1, resulting in the release of 4EBPs from eIF4E, thereby facilitating cap-dependent mRNA translation [25]. It has been reported that activation of mTORC1 enhances PD-L1 expression at the translational level [26]. A novel finding is that ALA (2 mM) could significantly suppress the phosphorylation of p70S6K and 4EBP1 (Figure 6A), suggesting that ALA may inhibit mTORC1-mediated protein synthesis. In response to Rapamycin, a mTORC1 inhibitor, IFN-γ-induced expression of p-p70S6K, p-4EBP1, HIF-1α, and PD-L1 was greatly inhibited (Figure 6B). Similarly, treatment with PF4708671 (20 µM), a p70S6K inhibitor, significantly suppressed the levels of IRF-1, c-Myc, and PD-L1 in a dose-dependent manner (Figure 6C). Accordingly, ALA-mediated inhibition of mTORC1/p70S6K/4EBP1-dependent protein synthesis is an important mechanism responsible for the decreased protein expression of PD-L1 and its upstream regulators.

### 3.7. ALA Inhibits GSK3β Activation and Its Regulated Processes

**Figure 7:**
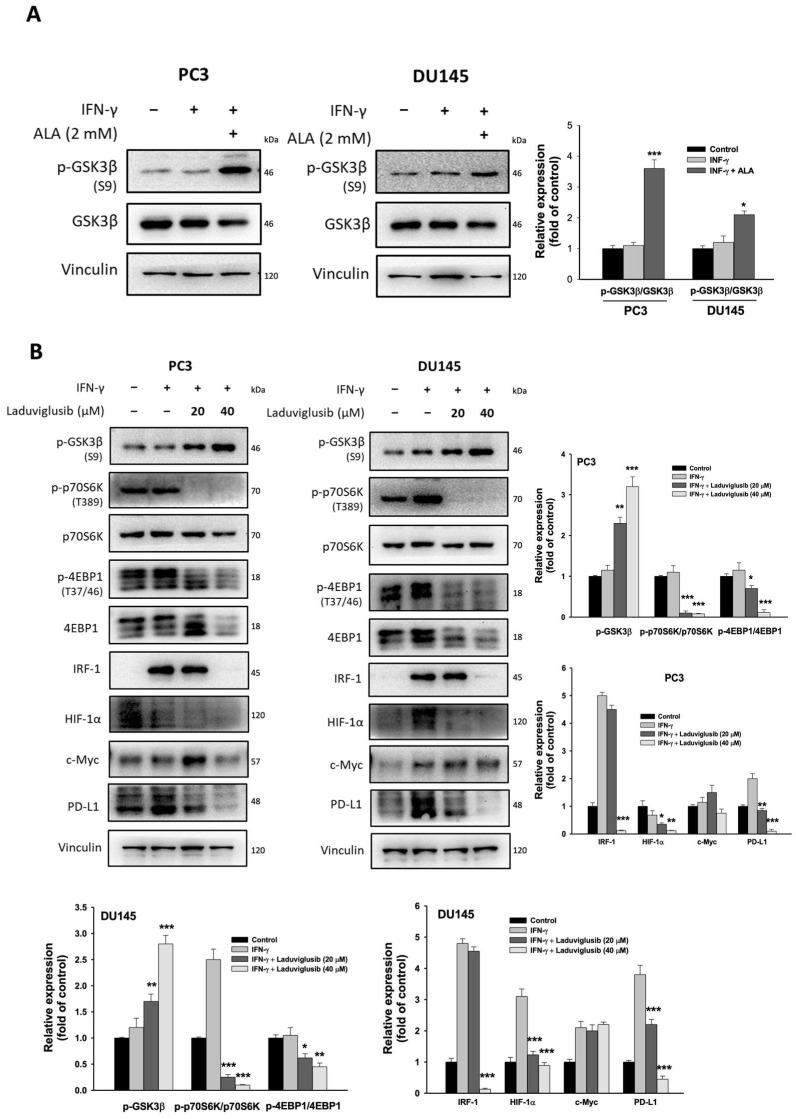
Effects of ALA on GSK3β activity and its regulated processes. (**A**) The PC3 and DU145 cells were pretreated with or without ALA (2 mM) for 1 h and then incubated for 6 h in the presence or absence of IFN-γ. The expression of p-GSK3β and GSK3β was determined. (**B**) The PC3 and DU145 cells were pretreated with or without Laduviglusib for 1 h and then incubated for 6 h in the presence or absence of IFN-γ. The relative proteins were determined. Results are expressed as mean ± S.E.M. * *p* < 0.05, ** *p* < 0.01, *** *p* < 0.001 vs. IFN-γ-treated alone cells.

GSK3β can promote mTORC1-regulated protein synthesis by directly phosphorylating p70S6K and 4EBP1 [27,28]. We found that ALA (2 mM) significantly elevated phosphorylated GSK3β (Ser9), an inactive form of GSK3β (Figure 7A). Inhibition of GSK3α/β with Laduviglusib (40 μM) markedly reduced the phosphorylation of p70S6K and 4EBP1, along with decreased protein levels of IRF-1, HIF-1α, and PD-L1, without affecting c-Myc expression (Figure 7B). These findings indicate that ALA-mediated inhibition of GSK3β activity attenuates mTORC1-dependent translation of IRF-1, HIF-1α, and PD-L1.

### 3.8. ALA Inhibits ROS Production and Its Regulated Processes

**Figure 8:**
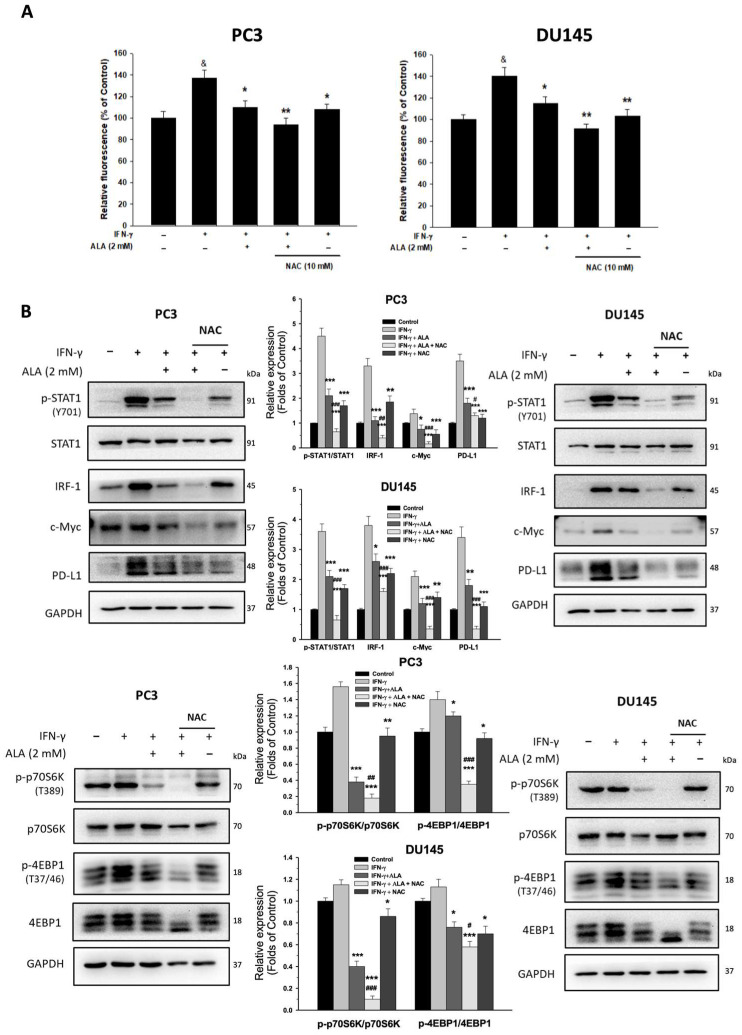
Effects of ALA and NAC on ROS production and downstream gene expression. (**A**) The PC3 and DU145 cells were pretreated with or without ALA (2 mM) or combined treatment with NAC (10 mM) for 1 h and then incubated for 6 h in the presence or absence of IFN-γ. The ROS production was measured. (**B**) The PC3 and DU145 cells were pretreated with or without ALA (2 mM) or combined treatment with NAC for 1 h and then incubated for 6 h in the presence or absence of IFN-γ. The expression of target proteins was determined. Results are expressed as mean ± S.E.M. ^&^ *p* < 0.05 vs. Control, * *p* < 0.05, ** *p* < 0.01, *** *p* < 0.001 vs. IFN-γ-treated alone cells. ^#^ *p* < 0.05, ^##^ *p* < 0.01, ^###^ *p* < 0.001 vs. IFN-γ- and ALA-treated alone cells.

It is considered that excessive ROS is a key inducer for PD-L1 expression in cancer cells [15]. As shown in Figure 8A, ALA (2 mM) reduces ROS production in IFN-γ-stimulated PC3 and DU145 cells. Treatment with N-acetylcysteine (NAC, 10 mM), an ROS scavenger, significantly inhibited STAT1 phosphorylation and the expression of IRF-1, c-Myc, and PD-L1, as well as the phosphorylation of p70S6K and 4EBP1, compared with cells treated with IFN-γ plus ALA alone (Figure 8B). Co-treatment with ALA (2 mM) and NAC further attenuated ROS generation and the expression of these target proteins (Figure 8B). Collectively, the antioxidant activity of ALA contributes to PD-L1 downregulation by suppressing STAT1 activation and the expression of IRF-1 and c-Myc and mTORC1-dependent translational signaling.

### 3.9. ALA Enhances the Tumor-Killing Activity of T Cells

**Figure 9:**
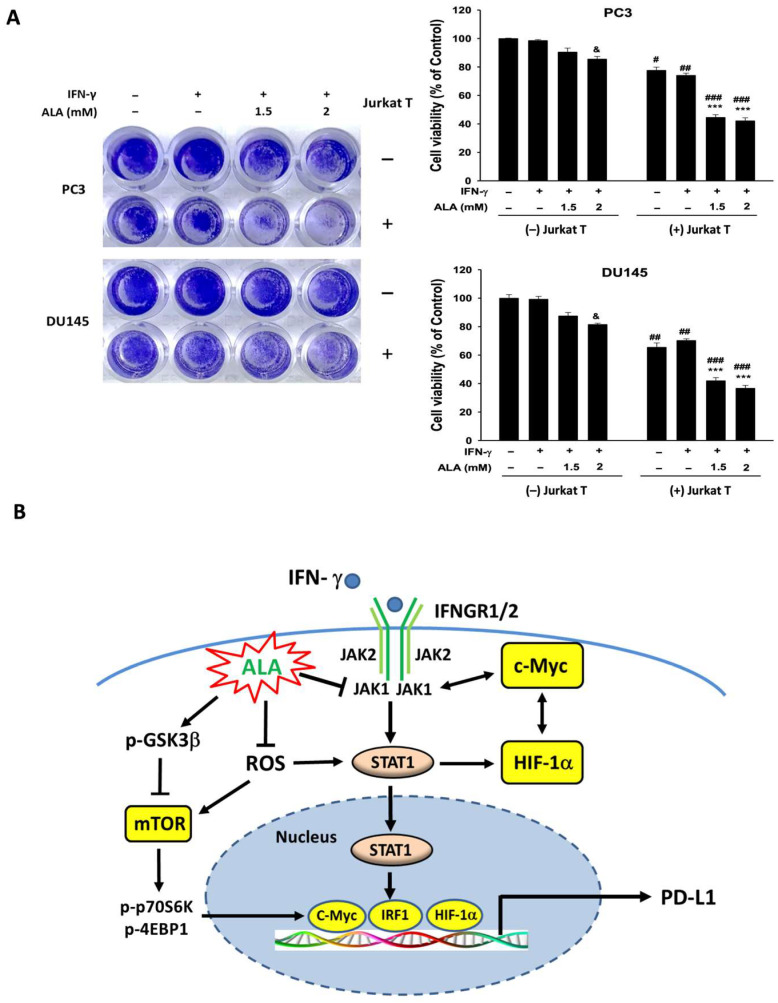
Effects of ALA on tumor-killing activity of T cells. (**A**) The PC3 and DU145 cells treated with or without ALA (1.5, 2 mM) for 1 h followed by addition of IFN-γ or not for 24 h. Then, the cells were washed and then the activated Jurkat T cells or medium only were co-cultured with PCa cells for 24 h. After the Jurkat T cells were removed, the morphology changes of cells were evaluated and the cell viability of PCa cells was determined by CCK-8 assay. (**B**) The proposed schematic illustrates that ALA inhibits PD-L1 expression in IFN-γ-stimulated PC3 and DU145 cells through multiple mechanisms. ALA suppresses JAK1/STAT1/IRF-1 signaling, c-Myc and HIF-1α expression, and GSK3β/mTOR/p70S6K/4EBP1-dependent protein translation, all of which contribute to the reduction in PD-L1 expression. Notably, a crosstalk exists among c-Myc, HIF-1α, and the JAK1/STAT1/IRF-1 signaling pathway. Furthermore, ALA-mediated attenuation of ROS production inhibits STAT1/IRF-1 activation, c-Myc expression, and mTOR-regulated translational processes. Collectively, ALA regulating these interconnected pathways ultimately downregulates PD-L1 expression, thereby enhancing T-cell-mediated tumor cytotoxicity. The arrows indicate activation, and the blunt-ended lines indicate inhibition. The bidirectional arrows indicate a mutual activation. Results are expressed as mean ± S.E.M. ^&^ *p* < 0.05 vs. IFN-γ-treated alone cells. *** *p* < 0.001 vs. IFN-γ- and T-cell-treated alone cells. ^#^ *p* < 0.05, ^##^ *p* < 0.01, ^###^ *p* < 0.001 vs. respective groups without T cells.

Binding of PD-L1 to PD-1 on T cells reduces the activation and cytotoxic function of T cells, thereby promoting tumor immune evasion [29]. To evaluate whether ALA enhances T-cell-mediated cytotoxicity, the co-culture system of PCa cells and Jurkat T cells was employed. Treatment with ALA (2 mM) alone only caused slight cell death of PCa cells. In contrast, in the co-culture system, ALA (1.5 or 2 mM) markedly increased PCa cell death (Figure 9A). These findings indicate that ALA may enhance T-cell-mediated antitumor cytotoxicity, thereby improving the efficacy of tumor immunotherapy.

## 4. Discussion

PCa is the second most common malignant tumor among men and the fifth leading cause of cancer-related deaths in men globally [30]. Recently, immunotherapy targeting the PD-L1/PD-1 pathway to prevent immune escape has become a promising therapeutic strategy in the treatment of tumors, particularly in solid tumors. Various PD-L1 inhibitors, predominantly monoclonal antibodies, have been applied in clinical practice. However, cancer patients often develop resistance to anti-PD-L1 immunotherapy with a response rate below 20%, which strongly limits the therapeutic efficacy and clinical application [31,32]. CRPC exhibits higher PD-L1 expression and lower responsiveness to immunotherapy compared with primary PCa [32], suggesting that PD-L1 may be an effective target to improve immunotherapy outcomes, particularly in CRPC. In the present study, we demonstrate that ALA significantly inhibits PD-L1 expression in CRPC-mimicking PC3 and DU145 cells by suppressing the JAK1/STAT1/IRF-1 signaling pathway, the expression of c-Myc and HIF-1α, as well as ROS production, thereby enhancing T-cell-mediated cytotoxicity against PCa cells.

Upon IFN-γ stimulation, the JAK1/STAT1/IRF-1 cascade is activated, leading to PD-L1 induction and ultimately promoting immunosuppressive tumor microenvironment [5]. Treatment with ALA for 0.5 or 1 h greatly inhibited IFN-γ-induced phosphorylation of JAK1/2 and STAT1 and the expression of IRF-1 and PD-L1 accompanied by attenuation of the nuclear accumulation of p-STAT1, IRF-1, and c-Myc. Thus, the JAK1/STAT1/IRF-1 pathway may be an early target of ALA, which subsequently modulates downstream gene expression. Pharmacological inhibition of this pathway greatly suppressed PD-L1 expression. Interestingly, treatment with IRF-1 inhibitor also reduced JAK1/STAT1 phosphorylation, suggesting that there is a positive feedback loop between IRF-1 and JAK1/STAT1 cascade.

c-Myc and HIF-1α are key translational factors for promoting PD-L1 expression and tumor progression in various tumor types, including CRPC [13,33,34]. We found that ALA treatment markedly suppressed c-Myc expression in IFN-γ-stimulated PC3 and DU145 cells. Inhibition of c-Myc activity significantly reduced the expression of HIF-1α and PD-L1 and the p-JAK1/p-STAT1/IRF-1 cascade. Interestingly, inhibiting JAK1/STAT1/IRF-1 pathway also attenuated c-Myc and HIF-1α expression. Moreover, addition of HIF-1α inhibitor (GN44028) significantly reduced PD-L1 and c-Myc expression, suggesting that inhibition of HIF-1α expression by ALA may account for the decreased PD-L1 expression. These findings indicate that there is a complex regulatory loop among c-Myc, HIF-1α, and the JAK1/STAT1/IRF-1 cascade. Overall, ALA-mediated inhibition of PD-L1 expression is regulated by multiple mechanisms through suppression of the JAK1/STAT1/IRF-1 signaling pathway and the c-Myc- and HIF-1α-dependent processes.

Next, whether ALA affects the protein synthesis and degradation rate of PD-L1 was examined. The ubiquitin-mediated proteasomal degradation is reported to regulate PD-L1 protein stability [35]. However, co-treatment with MG132 failed to elevate PD-L1 protein level, suggesting that PD-L1 synthesis may be impaired. When protein synthesis was blocked by CHX, ALA did not alter the degradation rate of PD-L1. Accordingly, ALA-mediated inhibition of PD-L1 protein expression is likely attributed to decreased protein synthesis rather than increased degradation. In contrast, ALA enhances IRF-1 degradation, as evidenced by an elevation in IRF-1 expression upon MG132 treatment and acceleration of the degradation rate of IRF-1 in the presence of CHX.

The mTORC1 complex is a critical factor promoting protein translation through phosphorylation of two downstream effectors, p70S6K and 4EBP1 [25]. Given that ALA markedly inhibited the phosphorylation of p70S6K and 4EBP1, it is likely that ALA has an ability to reduce mTORC1-dependent translational processes. Treatment with rapamycin remarkably inhibited the phosphorylation of p70S6K and 4EBP1, accompanied by decreased expression of IRF-1, HIF-1α, and PD-L1. Similarly, addition of p70S6K inhibitor significantly suppressed the expression of IRF-1, c-Myc, and PD-L1. Notably, GSK3β also promotes protein synthesis through phosphorylation of the regulatory subunit Raptor of mTORC1, 4EBP1, and p70S6K [27,28]. Our results showed that ALA markedly reduced GSK3β activity. As expected, inhibition of GSK3β activity significantly suppressed the phosphorylation of p70S6K and 4EBP1, as well as the expression of IRF-1, HIF-1α, and PD-L1 in IFN-γ-stimulated PC3 and DU145 cells. Collectively, ALA-mediated inhibition of GSK3β/mTORC1/p70S6K-dependent protein synthesis is a crucial mechanism contributing to the downregulation of the expression of PD-L1 and its upstream regulators. However, inhibition of GSK3β did not alter c-Myc expression. Previous studies have indicated that activation of GSK3β phosphorylates c-Myc at Thr58, leading to proteasomal degradation of c-Myc [36]. Thus, inhibiting GSK3β activity may increase c-Myc protein stability, while it simultaneously reduces mTORC1-dependent c-Myc translation. The opposing effects may result in no net change in c-Myc protein level after treatment with GSK3β inhibitor. Since ALA reduced PD-L1 mRNA level, ALA-mediated inhibition of PD-L1 expression may largely result from attenuation of both transcription and protein translation.

In general, cancer cells maintain moderately elevated levels of ROS to promote tumor progression and immune evasion through activation of oncogenic signaling pathways. However, excessive ROS cause mitochondrial dysfunction and oxidative damage, leading to cancer cell death [37], indicating that ROS exert dual roles in cancer progression. The ALA, a naturally occurring antioxidant, has been reported to reduce ROS production and induce apoptosis in MCF-7 human breast cancer cells and SMMC-7721 human hepatoma cells [21,38]. In contrast, ALA also increases ROS production, thereby promoting apoptosis in HT-29 human colon cancer cells [39]. In addition, ALA can trigger apoptosis through elevation of mitochondrial ROS in lung cancer cells [20]. Collectively, whether ALA functions as an antioxidant or a pro-oxidant in cancer cells appears to be context-dependent, which is influenced by multiple factors, including dosage, cancer cell type, the metabolic status of cancer cells, specific stimuli, and combination with other treatments. Of note, excessive ROS are able to induce PD-L1 expression and immune evasion through activation of NF-κB and c-Myc [14,15]. In the present study, we demonstrated that ALA reduced ROS production in IFN-γ-stimulated PC3 and DU145 cells. Treatment with NAC significantly inhibited the phosphorylation of p70S6K, 4EBP1, and STAT1, as well as the expression of IRF-1, c-Myc, and PD-L1. Co-treatment with ALA and NAC further reduced ROS production and the expression of these regulators. Thus, ALA-mediated attenuation of ROS production results in decreased PD-L1 expression via suppression of the levels of p-STAT1, IRF-1, c-Myc, and p70S6K, as well as p70S6K/4EBP1-dependent protein synthesis.

When PD-L1 binds to PD-1 receptors on T cells, it causes T-cell exhaustion and dysfunction, leading to tumor immune evasion [40]. Notably, treatment with ALA (2 mM) only induced mild cell death in IFN-γ-stimulated PC3 and DU145 cells, while, in the PCa-Jurkat T-cell co-culture system, ALA markedly increased cancer cell death. Since Jurkat cells are an immortalized human T-cell acute leukemia line, they do not fully represent the physiological heterogeneity and regulatory complexity of primary T cells. Thus, ALA-enhanced T-cell-mediated antitumor cytotoxicity observed in Jurkat cells may not accurately reflect the immune responses in vivo. Future studies using primary T cells and in vivo tumor model are required to determine the physiological relevance and therapeutic potential of ALA in cancer immunotherapy. Taken together, we demonstrated for the first time that ALA inhibits PD-L1 expression through multiple mechanisms, including suppressing JAK1/STAT1/IRF-1 cascade, the expression of c-Myc and HIF-1α, GSK3β activity, mTOR/p70S6K/4EBP1-dependent protein translation, and ROS production in IFN-γ-stimulated PC3 and DU145 cells. Consequently, ALA may enhance T-cell-mediated cytotoxicity against PCa cells.

## 5. Conclusions

ALA may be a potential agent to augment antitumor immunity through inhibition of PD-L1 expression, thereby preventing resistance to immunotherapy and tumor progression, particularly in CRPC.

## Data Availability

The original contributions presented in this study are included in the article. Further inquiries can be directed to the corresponding author.
